# Exploring LCME’s New USMLE Norms of Accomplishment: Medical School Self-Reflection to Support Continuous Quality Improvement

**DOI:** 10.1007/s40670-025-02488-4

**Published:** 2025-09-19

**Authors:** Mark Grichanik, Rivka C. Stone

**Affiliations:** 1 Educational Measurement Unit, David Geffen School of Medicine at the University of California, Los Angeles, Los Angeles, CA USA; 2 Elite Medical Prep, New York, NY USA; 3 Dr. Phillip Frost Department of Dermatology and Cutaneous Surgery, University of Miami, Miller School of Medicine, Miami, FL USA

**Keywords:** LCME accreditation, Continuous quality improvement (CQI), USMLE performance, Root cause analysis, Academic advising, Program evaluation

## Abstract

The Liaison Committee on Medical Education has introduced new norms for United States Medical Licensing Examination (USMLE) performance. Improving USMLE outcomes is a complex intervention that requires careful deliberation of tradeoffs, the coordination of many people and systems, and the marshalling of significant resources, so it is important that any increased attention to USMLE processes is approached strategically through a continuous quality improvement lens. We explore the potential implications of these new standards and present a self-reflection tool, inspired by the Ishikawa method, designed to help schools guide their USMLE-related initiatives by systematically considering possible root causes of below-target USMLE performance.

In response to guidance from the US Department of Education (DOE) [1], the Liaison Committee on Medical Education (LCME), the accrediting body for allopathic medical schools in the USA, recently established national norms of accomplishment for medical education programs to include metrics related to United States Medical Licensing Examination (USMLE), program attrition, and the National Resident Matching Program (NRMP) Match rate [2]. The USMLE is a three-step standardized exam required for medical licensure in the USA. The first two steps are completed prior to the start of graduate medical education (i.e., a residency program). Step 1 focuses on assessing the understanding and application of basic science to the practice of medicine. Step 2 Clinical Knowledge (CK) focuses on assessing the knowledge, skills, and understanding of the clinical sciences. The NRMP administers the Match, a program that pairs medical school graduates with residency program in the USA.

The new LCME norms of accomplishment were published in October 2023 and apply to schools with accreditation survey visits in the 2023–2024 academic year and onward. Housed under Standard 8, which governs curricular management, evaluation, and enhancement, the new norms outline specifications related to the evaluation of educational program outcomes, as follows:

Determination of performance in Element 8.4 (evaluation of educational program outcomes) includes a consideration of whether medical education program performance in the specific area in each year of the most recent two-year period, is outside of the following aggregate national performance data:

- **USMLE pass rate in Step 1** below 85%, which is 10% below the average pass rate over the most recent two years (95%)
- **USMLE pass rate in Step 2 CK** below 89%, which is 10% below the average pass rate over the most recent two years (99%)
- **Total percent attrition** during each of the last two academic years of 5% or greater per year (average total percent attrition during the most recent academic years is 1% per year)
- **Initial residency Match rate **of 83%, which is 10 percentage points below the average Match rate over the most recent two years (93%) [2].

Among these three areas, the attrition and Match rate criteria are themselves multifaceted phenomena impacted by many factors that may be outside of the school’s direct locus of control. Given their complexity and importance, they warrant comprehensive exploration outside the scope of this piece. In contrast, we maintain that a school can have the greatest *direct* impact in helping students achieve favorable outcomes in USMLE performance. Moreover, improving cohort- and student-level USMLE performance through institutional self-reflection and targeted interventions has the potential to also benefit attrition and Match outcomes. We therefore focus this monograph on the USMLE-related standards.

Although the LCME collects a vast amount of quantitative data from schools throughout the accreditation process, it is rare for them to establish specific thresholds for performance[3]. The introduction of new USMLE performance standards concretely defines a target outcome (the “*what*”) but allows flexibility in *how* a school might get from a current state to a target state. Therefore, our primary aims are (1) to forecast the potential implications of these new USMLE norms, and (2) to provide medical schools with a set of useful tools that they can use to guide their continuous quality improvement (CQI) initiatives in the USMLE performance space and satisfy the LCME benchmarks. We also discuss how USMLE considerations can have impacts on a medical school’s educational approaches and strategic priorities well beyond accreditation. As such, using this self-reflection as a tool to stimulate discussion about curriculum design, resource alignment, and student support can also benefit medical schools, even if they are already meeting the LCME benchmarks.

## Potential Implications for Medical Schools

USMLE performance already plays an outsize role in shaping outcomes for medical students. According to the most recent survey of residency program directors participating in the NRMP, 90% of program directors view a passing score on USMLE Step 1 as an important factor in considering which candidates to interview [4]. Furthermore, program directors who cited it as important assigned it a mean importance rating of 4.5 on a scale of 1 (not at all important) to 5 (very important). As such, although the USMLE program was “not designed to be a primary determinant of the likelihood of success in residency,” [5] it nonetheless is widely used for residency selection.

First-attempt USMLE Step 1 pass rates for examinees from US and Canadian medical schools have been on a downward trajectory over the past 4 years [6], which has already magnified attention given to USMLE performance at many medical schools [7]. The LCME’s standards are potent catalysts for shaping the policies and practices of their constituencies [8], and schools historically devote significant resources to processes and metrics that are the subject of regulatory scrutiny by the LCME [9]. There is already an increased trend by medical schools to commit personnel to systematically monitor institutional effectiveness through an accreditation lens, as evidenced by establishment of professional groups such as the Accreditation Professionals and Quality Improvement (APQI) [10]. Putting these factors together, we anticipate that the introduction of new USMLE performance standards in the LCME accreditation process will *increase* the amount of energy that medical schools expend on USMLE-related monitoring and programming.

As medical schools turn their attention toward the USMLE standards, it is unclear exactly how many may be performing below the LCME’s espoused minimum benchmark, since the National Board of Medical Examiners (NBME), which administers the USMLE program, does not publish school-level distributions of performance. The norm for USMLE performance at the school level is the mean first-attempt performance of examinees, which nationally was 93% for Step 1 in 2023 and 98% for Step 2 CK in 2022–2023 [6]. Medical schools are likely already motivated to align closely with the national average and adhere to the performance levels of peer institutions, and we posit that these new standards will universally heighten scrutiny of cohort-level USMLE performance. This further supports our overall premise that the mere inclusion of USMLE performance as an LCME standard, in general, and the norms of accomplishment, specifically, have the potential to change the behavior of schools.

## The Complexity of USMLE Interventions for Medical Schools and Their Students

Crafting USMLE interventions is challenging work for schools because it is difficult to maintain equilibrium among the many interdependent and often competing factors that can impact outcomes. For example, a school’s selection strategy during admissions can indirectly impact the likelihood of first-attempt USMLE pass rates [11, 12], as can a school’s curricular content [13], curricular structure [14], and the availability of student support programs and resources [15].

At the student level, a successful attempt at USMLE requires synthesizing domain knowledge in the basic and clinical sciences (i.e., content domain) and developing skills in study and exam strategy (i.e., process domain). Although success ultimately relies on the motivation and ability of the individual, schools can play a significant role by providing targeted resources that will enable students to perform their best (e.g., study resources, individualized tutoring, dedicated preparation time). Schools can quickly realize the dividends of individual-level interventions and avert a significant amount of USMLE failures. However, student-level interventions are reactive, tend to be resource-intensive, and can be psychologically and materially burdensome for individual students and the educational allies who support them.

At the cohort level, schools can proactively shape curricula and policies that can maximize the probability that *groups* of medical students will have the best chances of passing the USMLE exams. Schools might “invite” students to learn in a curriculum that is thoughtfully aligned with the USMLE and to create USMLE promotion policies that will interact synergistically with a school’s other strategic initiatives (e.g., early clinical exposure). Such interventions are complex, but when executed effectively can minimize the amount of time required to work with individual students and can create a sense of confidence in the student body and the medical school faculty and administration.

## Medical School Self-Reflection to Support CQI in USMLE Outcomes

The LCME requires that “a medical school engage in ongoing strategic planning and continuous quality improvement (CQI) processes” (Element 1.1) [2], and further requires that the medical school report on elements chosen for monitoring in a structured manner using the Data Collection Instrument (DCI) [3]. We suspect that schools whose USMLE metrics are between the new benchmark and the national norm would be self-motivated to engage in CQI initiatives, and that schools with performance below the minimum benchmark during an accreditation visit will receive an adverse status classification for Element 8.4 [16] and be formally compelled to include USMLE performance in their CQI processes. However, we propose that even those schools already meeting USMLE benchmarks will also benefit from utilizing this reflection as a tool to stimulate discussion about curriculum design, strategic planning, resource alignment, and student support, given that USMLE considerations impact — and are impacted by — a school’s strategy and priorities.

To this end, we now address the medical school personnel responsible for USMLE-focused CQI. Because USMLE challenges and interventions are multifactorial, we recommend using a multidisciplinary, team-based approach that includes perspectives from students, staff, faculty, and administrators from across the medical education enterprise. A few key units or personnel that your school may engage, depending on your specific challenge, include representatives from accreditation, assessment and evaluation, curricular affairs and student academic support, student affairs, curriculum committee, course or clerkship directors, behavioral health, graduate medical education, registrar, finance, project management, and information technology.

**Fig. 1:**
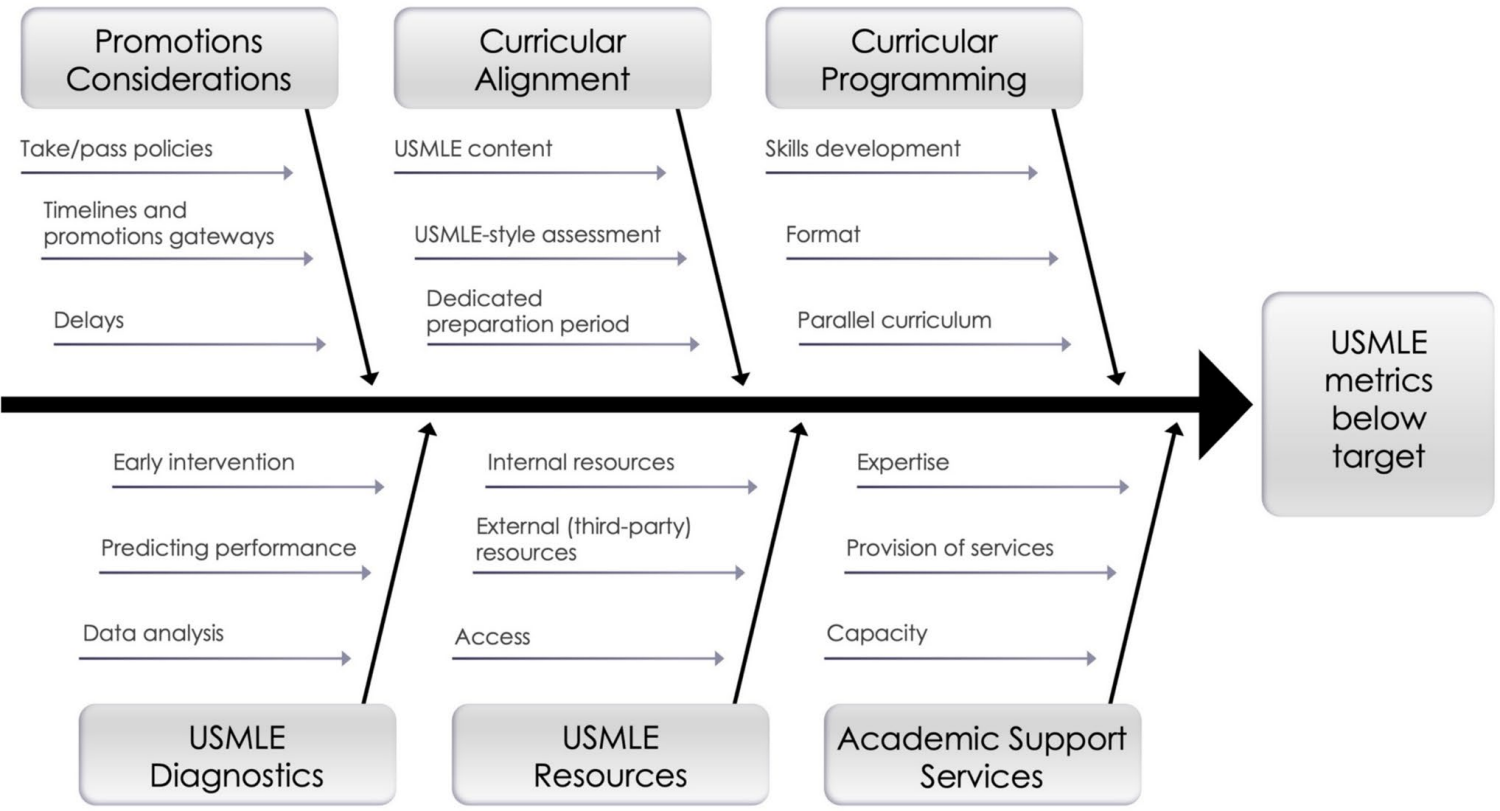
Fishbone/Ishikawa diagram summarizing potential causes (sub-branches) of below-target USMLE performance organized into six major domains (branches)

We outline a series of reflection questions that your medical school team can use to determine its current compliance with the new USMLE norms of accomplishment and to reflect on a variety of domains that may be impacting exam readiness. The logic of this self-reflection employs the fishbone or Ishikawa diagram method [17], which has been successfully applied to determine the root causes of poor exam performance in the medical education space [18] (Fig. 1). Suboptimal school-level USMLE performance is the “problem” at the head of the fish and is broadly framed as “USMLE metrics below target.” After clearly articulating a USMLE performance concern, which will be school-specific (Stage 1), the causes of these USMLE performance issues can be carefully examined through discussion-based self-reflection (Stage 2). We present potential contributing factors (“sub-branches”), which we sort into a list of six broad domains (“branches”) that may underlie or contribute to below-target cohort-level USMLE performance: promotions policies, curricular alignment, curricular programming, diagnostic practices, provision of study resources, and academic support services. After completing this self-reflection, your medical school team is encouraged to use formal data-driven methods to test the causes identified as most probable, move to implement corrective actions, monitor the interventions to see if changes led to improvement, and adjust them accordingly.

### Stage 1: Defining the Problem

The first stage in this self-reflection is to generate your medical school’s precise problem statement. This process begins with analyzing your organization’s USMLE data against the LCME’s USMLE norms of accomplishment, as follows:

1. Complete LCME DCI Table 8.4–3 [3] and LCME DCI Table 8.4–4 [3], which summarize the number of examinees as well as school-specific and national pass rates for Step 1 and Step 2CK, respectively, over the last 3 years. If the school’s passing percentage for first-time USMLE Step 1 test takers is below 85% for either of the last 2 years, or below 89% for Step 2 CK, the school is below the LCME’s national norm of accomplishment.
2. Generate your initial problem statement. For example, “Academic year 2025 USMLE Step 1 scores are below the LCME norm of accomplishment.”
3. Conduct subgroup analyses of USMLE pass rates and account for USMLE scheduling status (see Table 1) to get a more nuanced understanding of the problem. Organize this table by differentiating your student body into subgroups that you suspect might have meaningful differences in performance. For example, you may want to analyze by matriculation year if your school has recently gone through a substantial curriculum reorganization, or you may want to analyze by specialty programs (e.g., Pathway 1 students vs. Pathway 2 students). Is each subgroup where the school expects them to be relative to the USMLE timelines outlined for that class? Are there meaningful differences in the percentages of students who have attempted or passed the USMLE exam?
4. Refine your problem statement according to your subgroup analyses. For example, “Only 70% of students in the cohort that matriculated in 2028 have sat for Step 1 by the time we suggested, and only 80% of those who took the exam passed.”

**Table 1:** Summarize USMLE Step 1 or Step 2 CK attempts by subgroup to analyze meaningful exam states leading up to a first-attempt score

Subgroup	% not yet scheduled	% scheduled	% awaiting scores	% passed	% failed
Subgroup 1					
Subgroup 2					
Subgroup 3					
Subgroup 3					

### Stage 2: Exploring Potential Causes of USMLE Metrics That Are Below Target

The second stage of this process is to explore the underlying causes that may contribute to the problem you outlined in Stage 1. The Ishikawa/fishbone diagram shown in Fig. 1 and described in detail below will guide you in reflecting as to how each of the six domain “branches” and the causes within them (“sub-branches) may contribute to your problem statement. Note that your medical school team may identify several potential causes contributing to the problem or that there are interactions among multiple causes. Read the introductory paragraph for each branch and consider your responses to the questions posed for each sub-branch:

### Branch 1: Promotions Considerations

Use this branch to explore your medical school’s macro-level curricular policies that may interact with your students’ USMLE preparation. Consider that policies governing the timing of USMLE attempts have a profound effect on USMLE outcomes as well as student progress through the medical school curriculum [19, 20]. For example, requiring students to sit for USMLE Step 1 as an internal promotion gateway before students have had a sufficient opportunity to acquire and consolidate sufficient knowledge in the foundational sciences can lead to delays and/or lower pass rates.

- **Take/Pass Policies**: Do students have to take and/or pass USMLE Step 1 and Step 2 CK for advancement and/or graduation (see LCME DCI Table 8.4–1 [3])? What is the rationale for these requirements? How might your program be affected if you changed any of these requirements to be more stringent or more permissive?
- **Timelines and Promotions Gateways**: What are the timelines in the promotions policy that outline when the school recommends or requires students to take or take-and-pass each of the USMLE exams [14, 19–23]? What is your school’s rationale for creating these timelines? Do these timelines put students in the strongest position to sit for the exam when considering the content and cadence of the curriculum? What would be the effect of adding, removing, or changing these timelines on the program?
- **Delays**: Do students typically sit for the exam when you recommend or require an attempt (i.e., what is your delay rate)? What are the policies and procedures that govern USMLE delays or re-attempts? Does your school prospectively collect and store data in the student information system about reasons for USMLE delays? Historically, what have been the reasons for delays? How can students request a delay? By whom and how quickly is the delay request adjudicated? What are the internal consequences of a delay (e.g., not allowed to start next phase of curriculum, financial aid implications)? Is there formal infrastructure (e.g., independent study courses, academic support) set up for students who delay or need to re-attempt?

### Branch 2: Curricular Alignment

This branch will help you explore whether there is synergy between USMLE needs and other activities (e.g., preparing students for clerkships) within your formal curriculum. Considering the vast scope of physician education, many priorities compete for curricular footprint, including USMLE preparation. The degree to which USMLE preparation aligns with your medical school’s mission-specific curricular priorities can impact the way students spend their time [24].

- **USMLE Content**: What role does the USMLE Content Outline [25] play in the development of curricular objectives for your school? How familiar are course directors and curriculum committee members with the USMLE Content Outline? What is the balance between “boards and wards” in curriculum mapping? What is the general attitude of curriculum planners at the school regarding USMLE preparation or “teaching to the test” [26, 27]?
- **USMLE-Style Assessment:** How prominent are “USMLE-style” questions in your school’s internal assessments in both format and difficulty [28]? How much training do internal item writers have in developing USMLE-style questions? In considering how students spend their time studying, how are they incentivized to perform well on internal exams versus USMLE exams?
- **Dedicated Preparation Period**: Does the curriculum provide students with a dedicated preparation period? If so, how long is this dedicated period? Has the school collected any feedback from students about the perceived utility of this time?

### Branch 3: Curricular Programming

Medical schools can design the degree to which USMLE preparation is a part of the formal curriculum or a co-curricular activity. This branch explores the types of development your medical school provides.

- **Skills Development**: What programming or resources does your school offer to support students in the following areas?
  - Exam overview (e.g., role of USMLE, exam format, when and how to schedule)
  - Skills related to exam preparation and testing (e.g., resource selection, study plan development and revision, exam pacing)
  - Review of USMLE content (e.g., knowledge of the cardiovascular system)
  - Exam-related wellness and stress management [29]
- **Format:** In what formats is USMLE-related programming delivered (e.g., workshops vs lectures, asynchronous vs. synchronous, in-person vs virtual, small-group vs. whole-group)? Is the programming elective or required? What is the rationale underpinning the timing of when this programming is delivered? Who is responsible for the planning and delivery of this programming?
- **Parallel Curriculum**: What tend to be attitudes regarding the “parallel” curriculum” (i.e., students using third-party resources) [30]? Are these resources welcomed and woven into the formal curriculum, or is their use discouraged?

### Branch 4: USMLE Diagnostics

This branch explores whether your school gathers data to predict USMLE performance at various stages, and how you use that data to individualize the preparation of students or cohorts. Consider that there are meaningful quantitative predictors of USMLE performance well upstream of a USMLE attempt and additional predictors that become useful as students get closer to an attempt.

- **Early Intervention:** Has your school conducted any studies to identify local predictors of USMLE performance? These may include pre-matriculation metrics (e.g., MCAT scores) [11], internal assessments (e.g., summative exams) [31], or student status information (e.g., students experiencing challenges in their life outside of school) [29]. How early can your school predict the likelihood of adverse USMLE outcomes? How quickly does your school intervene when a student likely to require additional support is identified?
- **Predicting Performance:** Does your school administer any NBME Comprehensive Basic or Clinical Science Exams (CBSE/CCSE) and/or provide vouchers for students to take the NBME Comprehensive Basic or Clinical Science Self-Assessment (CBSSA/CCSSA) [32]? When are these administered relative to other key landmarks (e.g., at the beginning of dedicated study)? Is this done only for select students or for the entire cohort? Do proctoring practices for these practice exams align with USMLE norms (e.g., timing, testing center protocol)?
- **Data Analysis**: How is performance on these assessments benchmarked? How is the data at the cohort level used to manage curricular programming? Is student-level data used to identify students who may need additional support? What performance information is shared with students (e.g., only individual data, cohort-level performance, historical data with predictive information)?

### Branch 5: USMLE Resources

There is a wealth of resources devoted to preparing students for USMLE exams. The resources vary in quality and students need to be trained in how to select appropriate tools, how to incorporate them effectively into their studying, and how to use them alongside the medical school curriculum. This branch explores access that your medical school provides to these tools and how you approach training your students to use them.

- **Internal Resources**: Does you school have any peer support resources [33]? These may include peer tutoring, organized study groups, or peer support groups. Who trains and supervises the peer tutors? How does the school know if the programming has been effective? Does your school develop internal resources to support USMLE preparation? These may include USMLE study plans that are mapped on top of the school’s curriculum, internally developed question banks, and content review resources. What training do students, faculty, and advisors receive in developing and utilizing internal resources effectively?
- **External (Third-Party) Resources**: Does your school provide or facilitate any third-party resources specifically designed to support students in USMLE preparation? [34–37] These may include (i) resources that help with study organization (e.g., tools that generate student-specific study plans); (ii) access to USMLE question banks [38]; (iii) resources that cover USMLE-related content (e.g., spaced-repetition/flashcard platforms, video series, review books) [37]; (iv) USMLE preparation or remediation programs (e.g., in-residence programs, virtual preparation courses); (v) professional USMLE 1:1 tutoring services [39].
- **Access**: What process does your school use to direct students to specific resources? If third-party resources are provided, are they purchased by the school, packaged into cost-of-attendance calculations for financial aid consideration, or supported using an alternative model? For resources purchased by students, do school administrators or student leaders organize initiatives to order in bulk and receive discounted pricing? Does your school monitor whether and how student and instructors are using the supported resources?

### Branch 6: Academic Support Services

Advisors from academic support offices can help ensure that students requiring additional support for USMLE preparation receive it from informed professionals at appropriate points. This branch explores the expertise and capacity your medical school devotes to support students in USMLE preparation and how advisors manage your students’ needs.

- **Expertise:** How do your advisors acquire and maintain expertise in USMLE preparation? How do they keep up with developments in exam content and study resources? Are there advisors who can cover all the programming, or does the school have individuals who specialize in specific areas (e.g., medical content tutoring vs study plan development)? What is the referral process for students requiring support beyond the advisor’s expertise?
- **Provision of Services:** How do advisors identify students requiring additional support? Can students initiate the request for additional support? Who manages communication and touchpoints with the students in need of additional support? What services are these students eligible for above-and-beyond the general student body? Once students are offered support, is their participation elective or required? Are these services provided to individual students or to small cohorts? Are there any special conditions that students must agree to before receiving additional support or resources?
- **Capacity:** Do academic advisors have sufficient capacity to support all students in need? Does that capacity align with surges in demand driven by intensive periods of USMLE preparation? What is the ratio of academic advisors to students? Can advisors access external resources for students requiring USMLE interventions that exceed the school’s internal capacity?

## Insights Regarding Best Practices

The six domains presented here (promotions policies, curricular alignment, curricular programming, diagnostic practices, provision of study resources, academic support services) and the potential causes contained within them were developed inductively. We initially referenced the robust medical education literature that examines the predictors of USMLE.

performance [11, 12, 14, 15, 31, 32] and interventions that may impact it [13, 19, 20, 24, 30, 33–37]. In addition, we leaned on expertise developed by consulting for dozens of medical schools to design customized programs that support their students in USMLE preparation. In our experience, schools that have been the most effective in optimizing USMLE outcomes share several traits. First, they create quantitative models composed of school-specific predictors of sentinel events (e.g., first-attempt USMLE failure) and then engage in early, ongoing surveillance of those predictors among their students. Second, they leverage high-quality exam preparation resources by teaching students how to use them and integrating them organically into or around their curriculum. Third, they proactively require that “at-risk” students engage in individualized coaching with knowledgeable USMLE experts within and/or outside their school. While there is no “one size fits all” intervention, we have observed that schools that implement these practices are most successful at achieving high pass rates, preserving student self-esteem, maximizing student self-efficacy and maintaining their momentum through the curriculum, and maximizing return on organizational resources.

## Conclusion

Medical schools are challenged by balancing many interdependent factors that can impact USMLE outcomes. With the LCME’s introduction of the new USMLE benchmarks, all accredited medical schools are now compelled to systematically report performance on USMLE Step 1 and Step 2 CK to remain in compliance. For some schools, this process will feel very familiar in that USMLE performance is already actively monitored and benchmarked as part of internal CQI, and interrogating compliance with LCME norms will naturally follow. For others, the norms may necessitate the establishment of new monitoring and intervention processes. Regardless of where a school currently stands vis-a-vis the new requirements, the complexity of USMLE-related interventions will become more evident as medical schools begin to dig deeper. We believe that it is at this critical juncture that the self-assessment tool presented in this work can be most helpful. There are no correct or incorrect answers to the questions being posed. Rather, as a root cause analysis tool, the *process* of interrogating these facets is what will generate the meaningful insights and conclusions that can be carried forward to guide improvement of USMLE interventions and resultant outcomes. The primary initial motivator for this exercise may be compliance with the LCME requirements to maintain accreditation. However, by thoughtfully and proactively undertaking this CQI-motivated self-reflection, schools may reap a valuable added benefit: a cohesive set of USMLE policies and procedures that reflect a school’s values and priorities to support the success of its students.
